# Simultaneous Inhibition of T Helper 2 and T Regulatory Cell Differentiation by Small Molecules Enhances Bacillus Calmette-Guerin Vaccine Efficacy against Tuberculosis*[Fn FN2]

**DOI:** 10.1074/jbc.M114.600452

**Published:** 2014-10-14

**Authors:** Debapriya Bhattacharya, Ved Prakash Dwivedi, Santosh Kumar, Madhava C. Reddy, Luc Van Kaer, Prashini Moodley, Gobardhan Das

**Affiliations:** From the ‡ Laboratory Medicine and Medical Sciences, College of Health Sciences, University of Kwazulu Natal, Durban 4001, South Africa,; § Yogi Vemana University, Kadapa 516003, India,; ¶ Department of Pathology, Microbiology and Immunology, Vanderbilt University School of Medicine, Nashville, Tennessee 37232, and; ‖ Special Centre for Molecular Medicine, Jawaharlal Nehru University, New Delhi 110067, India

**Keywords:** Cytokine, Immunotherapy, Mycobacterium tuberculosis, T Helper Cells, Vaccine

## Abstract

Tuberculosis affects nine million individuals and kills almost two million people every year. The only vaccine available, Bacillus Calmette-Guerin (BCG), has been used since its inception in 1921. Although BCG induces host-protective T helper 1 (Th1) cell immune responses, which play a central role in host protection, its efficacy is unsatisfactory, suggesting that additional methods to enhance protective immune responses are needed. Recently we have shown that simultaneous inhibition of Th2 cells and Tregs by using the pharmacological inhibitors suplatast tosylate and D4476, respectively, dramatically enhances *Mycobacterium tuberculosis* clearance and induces superior Th1 responses. Here we show that treatment with these two drugs during BCG vaccination dramatically improves vaccine efficacy. Furthermore, we demonstrate that these drugs induce a shift in the development of T cell memory, favoring central memory T (Tcm) cell responses over effector memory T (Tem) cell responses. Collectively, our findings provide evidence that simultaneous inhibition of Th2 cells and Tregs during BCG vaccination promotes vaccine efficacy.

## Teaser

**Background:** Immunological parameters induced by BCG and the requirement of immunologic responses for optimal vaccine efficacy is incompletely understood.

**Results:** Small-molecule inhibitors of Th2 and Treg cells promote BCG vaccine efficacy.

**Conclusion:** Immunomodulators enhance the capacity of the BCG vaccine to protect against tuberculosis.

**Significance:** Our studies reveal a simple and cost-effective approach to improve BCG vaccine efficacy.

## Introduction

Tuberculosis (TB)^3^ is the cause of two million deaths each year, which is the second highest cause of mortality from a single infectious disease worldwide (1). One-third of the global population is latently infected with *Mycobacterium tuberculosis*, waiting for the opportunity of perturbations in the immune response such as those induced by HIV infection (2) for reactivation. Thus, the vast reservoir for TB disease is alarming, and its epidemic is becoming a global public health emergency. Unfortunately, cost-effective and user-friendly therapy of TB is long overdue. Bacillus Calmette-Guerin (BCG) is the only TB vaccine presently available. It has been widely used throughout the world since its inception in 1921, and an estimated three billion people have received it. BCG is effective against disseminated and meningeal tuberculosis in young children. However, its efficacy against adult pulmonary TB varied dramatically between 0 and 80% in different populations depending on ethnicity and geographical locality (3). Recent studies have indicated that BCG-vaccinated animals mainly develop antigen-specific, CD4^+^ T cell effector memory (Tem) cells. Therefore, an apparent failure of BCG to induce significant numbers of central memory T (Tcm) cells may be an important contributing factor to its limited vaccine efficacy (4).

It is well known that *M. tuberculosis* survives and replicates within host cells by modulating T helper (Th) cell responses. Studies with patients and animal models have indicated that T cells are indispensable for anti-TB immunity. Resistant individuals mount *M. tuberculosis* antigen-specific Th1 responses, as determined by preferential T cell production of IFN-γ, lymphotoxin, and tumor necrosis factor-α (TNF-α) (5). Similarly, individuals defective in genes for IFN-γ or the IFN-γ receptor are highly susceptible to TB (6). Animal models of TB confirmed that *M. tuberculosis*-specific Th1 cells are indispensable for elimination of tubercle bacilli from the host (7). However, several studies have provided evidence that Th1 responses alone are not sufficient for protection against TB (8). Furthermore, mouse strains with enhanced susceptibility to *M. tuberculosis* infection induce progressive Th2 responses predominated by production of IL-4, IL-5, and IL-13 (9). Thus, Th2 responses might contribute to enhanced susceptibility to TB. This hypothesis was strengthened by the finding that IL-4-deficient mice are resistant to *M. tuberculosis* infection (10). Similarly, studies investigating the expression of cytokines in human granulomas of patients with advanced TB revealed increased IL-4 production (11). Interestingly, elevated Th2 responses have been noted in patients who failed to be protected from TB after BCG vaccination (12). Nevertheless it is clear that susceptibility to TB is not limited to individuals with enhanced Th2 cell responses. Another T cell subset, T regulatory (Treg) cells (CD4^+^CD25^+^FoxP3^+^ T cells), is expanded during the progression of TB and contributes to disease susceptibility (13). Antigen-specific Treg cells increased within 3 weeks of infection and were associated with an environment that increased bacterial burden (14) and inhibited the development of protective Th1 responses. Although the precise cytokine requirements for the differentiation of Treg cells remain unclear, it has been established that expression of the forkhead transcription factor FoxP3 is inducible by TGF-β. In a recent study we demonstrated that mice unable to mount Th2 and Treg cell responses (*i.e.* Stat-6^−/−^CD4-TGFβRIIDN mice) are highly resistant to *M. tuberculosis* infection (15). We further validated these data by small molecule-directed immunotherapy using suplatast tosylate ([3-[[4-(3-ehoxy-2-hydroxypropoxy)phenyl]amino]-3oxopropyl]dimethylsulfonium 4-methylbenzenesulfonate) and D4476 (4-[4-(2,3-dihydro-1,4-benzodioxin-6-yl)-5-(2-pyridinyl)-1H-imiodazol-2-yl]benzamide), which inhibit Th2 and Treg cell differentiation, respectively. Combined treatment with these agents rapidly decreased the bacterial burden in mice. This was associated with increased Th1 cell responses, as shown by a dramatic increase in IFN-γ-producing cells with a moderate increase in IL-17-producing cells and by the finding that this therapeutic regimen was not effective in T-bet-deficient animals that are unable to produce Th1 type immune responses (15). These observations suggested that combined inhibition of Th2 and Treg cell differentiation promotes protective immune responses in the host, which is in agreement with the concept that Th1 cells are necessary and sufficient for resistance against TB (16). As these compounds enhance host-protective immune responses, which successfully eliminate the harbored *M. tuberculosis* organisms, it is likely that this therapeutic modality induces long-lasting protective memory responses in the host.

These findings suggested that mounting Th1 responses while inhibiting Th2 and Treg responses should be beneficial in developing TB vaccines. We, therefore, tested this hypothesis using BCG. Our results showed that simultaneous inhibition of Th2 and Treg cell differentiation enhances the efficacy of BCG vaccination, which was associated with enhanced Th1 responses. Recent studies have indicated that attenuation of Tregs during BCG immunization increases the efficacy of BCG by enhancing the production of Th1 responses (17). Furthermore, studies suggested that the presence of IL-4 in the microenvironment corrupts the Th1 immune response (18). These authors also provided evidence that increased IFN-γ and IL-17 concentrations by means of inhibition of IL-4, IL-5, and IL-10 improve BCG vaccine efficacy. Here we have showed that inhibition of Th2 cells and Tregs promotes host-protective Th1 responses and thereby enhances BCG vaccine efficacy. Therefore, we observed a dramatic switch in the memory T cell response toward Tcm cell responses. Consistent with the central role Tcm cells play in host protection and vaccine efficacy, animals treated with these two inhibitors at the time of BCG vaccination exhibited significantly improved protection against *M. tuberculosis* infection. Therefore, this strategy holds promise for developing improved TB vaccines in humans.

## EXPERIMENTAL PROCEDURES

### 

#### 

##### Mice

BALB/c mice, either Thy1.1^+^ or Thy1.2^+^ (6–8 weeks of age), were initially purchased from The Jackson Laboratory. All animals were subsequently bred and maintained in the animal facility of the International Centre for Genetic Engineering and Biotechnology (ICGEB), New Delhi, India.

##### Immunization Studies and Treatment with Immunomodulators

Mice were immunized with BCG subcutaneously (1 × 10^6^ bacteria), and starting from the next day these animals were treated with D4476 (TGFβRI inhibitor) and suplatast tosylate (Th2 inhibitor) purchased from Tocris at 16 nmol/g of body weight for a total of 10 days. Mice were subsequently rested for 20 days. Mice were then challenged by the aerosol route with *M. tuberculosis* strain H37Rv, and organs were harvested for determination of bacterial burden at 60 days after infection.

##### Bacterial Infections

*M. tuberculosis* H37Rv strain was grown in Middlebrook 7H9 broth (BD Biosciences) containing 0.02% Tween 80 to mid-log phase at 37 °C for 3 weeks, then aliquoted and frozen at −80 °C until use. Viable bacterial number was determined on 7H11 agar plates (BD Biosciences) with oleic acid-albumin-dextrose-catalase (OADC) enrichment (BD Biosciences). Mice were infected via the aerosol route using the nebulizer compartment of an airborne infection apparatus. After 30 min of exposure the deposition of bacteria was ∼110 bacteria/lung, which was determined by plating the lung homogenates after 24 h of infection. The numbers of viable bacteria in the lung, spleen, and liver of different types of mice were followed at regular time intervals by plating serial dilutions of individual organ homogenates onto nutrient Middlebrook 7H11 agar and counting bacterial colony formation after 21 days of incubation at 37 °C.

##### T Cell Adoptive Transfer

For adoptive transfer experiments, Thy1.1^+^ mice were γ-irradiated (8 rads/s for 100 s) and rested for 1 day. CD4^+^ T cells, isolated from the lymph nodes of Thy1.2^+^ animals, were then adoptively transferred into the irradiated recipient mice (2 × 10^6^ cells per mouse). After 10 days recipient mice were challenged with H37Rv through the aerosol route.

##### FACS and Intracellular Cytokine Staining

For intracellular cytokine staining, cells were treated with 50 ng/ml phorbol myristate acetate and 500 ng/ml ionomycin in the presence of 10 μg/ml brefeldin A (Sigma) added during the last 6 h of culture. Cells were washed twice with PBS and resuspended in a permeabilization buffer (Cytofix/Cytoperm kit; BD Biosciences) and stained with the following fluorescently conjugated monoclonal antibodies: anti-mouse CD4 (clone GK1.5)-APC (eBioscience), anti-mouse CD4 (clone GK1.5)-PE-Cy5 (eBioscience), anti-mouse CD4 (clone GK1.5)-PE (eBioscience), anti-mouse CD4 (clone GK1.5)-FITC (eBioscience), anti-mouse CD8 (clone 53–6.7)-PE-Cy5 (eBioscience), anti-mouse CD44 (clone IM7)-PE (Biolegend), anti-mouse CD62L (clone MPL-14)-APC (eBioscience), anti-mouse CD25 (clone PC61)-APC (Biolegend), anti-mouse IFN-γ (clone XMG1.2)-APC (Biolegend), anti-mouse IL-4 (clone 11B11)-PE (Biolegend), anti-mouse IL-17A (clone TC11–18H10.1)-PE (Biolegend), anti-mouse FoxP3 (clone MF-14)-PE (Biolegend), anti-mouse FoxP3 (clone FJK-16s)-APC (eBioscience), anti-mouse TNF-α (clone MP6-XT22)-PE (Biolegend), anti-mouse IL-6 (clone MP5–20F3)-PE (Biolegend), anti-mouse IL-12 (clone C15.6)-PE (Biolegend), and anti-mouse IL-10 (clone JES5–16E3)-PE (Biolegend). Fluorescence intensity was measured by flow cytometry (FACS Calibur or FACS CantoII; BD Biosciences), and data were analyzed with FlowJo (Tree Star).

##### Cytokine Assay

Cytokines in the culture supernatant of splenocytes were assayed by a Luminex microbead-based multiplexed assay using commercially available kits according to the manufacturer's protocol (BioPlex, Bio-Rad).

##### Statistical Analysis

All data were derived from at least three independent experiments. Statistical analyses were conducted using SPSS10 software, and values are presented as the mean ± S.D. Significant differences between the groups were determined by analysis of variance followed by Tukey's multiple comparison test (SPSS software). A value of *p* < 0.05 was used as an indication of statistical significance.

## RESULTS

### 

#### 

##### Simultaneous Treatment with Th2 and Treg Cell Inhibitors Increases BCG Vaccine Efficacy

**FIGURE 1.**
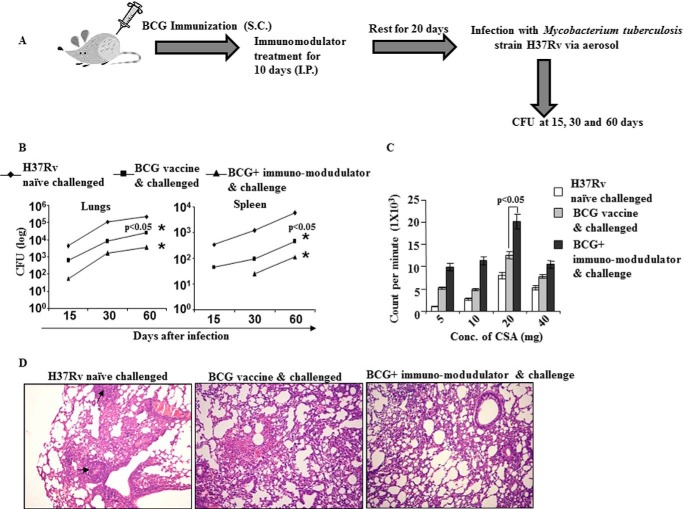
**Immunomodulators enhance the capacity of a BCG vaccine to reduce bacterial burden upon *M. tuberculosis* challenge.** *A*, schematic diagram of the immunization scheme, treatment with immunomodulators, and infection of BALB/c mice. *I.P.*, immunoprecipitate; *S.C.*, subcutaneous. *B*, after immunization BALB/c mice were treated daily with D4476 (TGFβRI inhibitor) and/or suplatast tosylate (Th2 inhibitor) at 16 nmol/g of body weight for a total of 10 days, and the mice were then rested for 20 days before aerosol challenge with a low dose of *M. tuberculosis* (H37Rv) (∼100 cfu). Bacterial burdens (cfu) were measured in lungs and spleens at 60 days post infection. Data represent the mean ± S.D. values of four mice per group per time point, and the experiment was repeated twice. *C*, proliferation of splenocytes in response to complete soluble antigen was measured by a [^3^H]thymidine incorporation assay. *CSA*, complete soluble antigen. *D*, photomicrographs (×10) of histological lung sections (6 μm) at 60 days after infection of the indicated mice, stained with hematoxylin and eosin. Results shown here are representative of three independent experiments.

To examine the effect of Th2 and Treg cell inhibitors on BCG-vaccinated mice, we immunized BALB/c mice with BCG and treated the animals with D4476 and suplatast tosylate, inhibitors of TGFβRI signaling and Th2 cell differentiation, respectively, for 10 days. These animals were subsequently rested for another 20 days and re-infected with *M. tuberculosis* strain H37Rv through the aerosol route. Organs were harvested, and the bacterial burdens were determined at different time intervals (Fig. 1*A*). We observed that co-treatment with these compounds drastically enhanced BCG vaccine efficacy, as determined by the significant reduction of bacterial loads in various organs (Fig. 1*B*). To obtain information regarding potential alterations in immune responses, we challenged spleen cells isolated from mice 60 days after infection with *M. tuberculosis*-derived complete soluble antigen and measured proliferative responses. We found that splenocytes from animals that received immunomodulators along with BCG exhibited superior proliferative responses (Fig. 1*C*). Histological studies further revealed that animals treated with immunomodulators along with BCG showed a dramatic reduction in the regions of the lungs containing granulomas (Fig. 1*D*).

##### Inhibition of Th2 and Treg Cell Differentiation during BCG Immunization Enhances CD4^+^CD44^hi^CD62L^hi^ T Cell Responses

**FIGURE 2.**
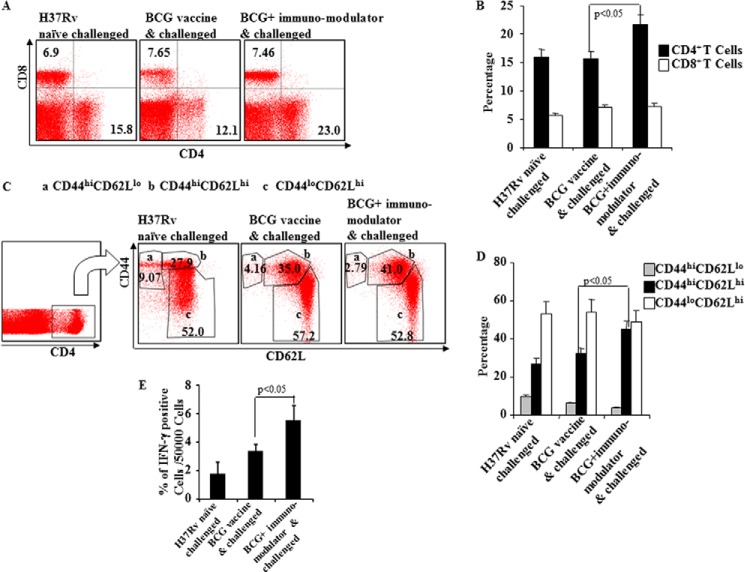
**Induction of multifunctional CD4^+^ T cells after BCG immunization and immunomodulator treatment of mice.** *A* and *B*, CD4^+^ and CD8^+^ T cell counts at 60 days post infection of immunized and immunomodulator-treated mice. *C* and *D*, expression of different surface phenotypes (*CD44^hi^CD62L^lo^*, *CD44^hi^CD62L^hi^*, *CD44^lo^CD62L^hi^*) by multifunctional CD4^+^ T cells. *E*, prevalence of IFN-γ-producing cells among CD44^hi^CD62L^hi^ cells at 60 days post infection of immunized and immunomodulator-treated mice. Results shown here are representative of three independent experiments.

To provide insight into the T cell response induced by the immunomodulators in BCG-vaccinated animals, we analyzed T lymphocytes from spleens of different experimental groups of animals. Phenotypic characterization revealed that CD4^+^ T cells in animals treated with suplatast tosylate and D4476 were increased significantly as compared with mice treated with BCG only (Fig. 2, *A* and *B*).

Vaccine efficacy is mostly dependent on the pool of Tcm (19), whereas BCG primarily induces Tem in lung, which might contribute to the limited efficacy of BCG, providing only short term protection (4). As inhibition of Th2 and Treg cell differentiation with pharmacological inhibitors promoted disease resistance, we determined the relative generation of Tem and Tcm cells in immunized animals. Phenotype analysis revealed that approximately half of all CD4^+^ T cells in the animals treated with the immunomodulators were CD44^hi^CD62L^hi^, indicating Tcm, which is significantly higher than in any other experimental group (Fig. 2, *C* and *D*). In contrast, the numbers of CD44^hi^CD62L^lo^ CD4^+^ T cells, which are the phenotype of Tem, were dramatically decreased in these animals (Fig. 2, *C* and *D*). To further determine the functional relevance of these cells, we determined IFN-γ production by these cells. We observed that the CD44^hi^CD62L^hi^ cells derived from mice treated with immunomodulators included significantly increased levels of IFN-γ-producing cells (Fig. 2*E*).

##### Th Subset Responses in BCG Immunized Mice Treated with Immunomodulators

**FIGURE 3.**
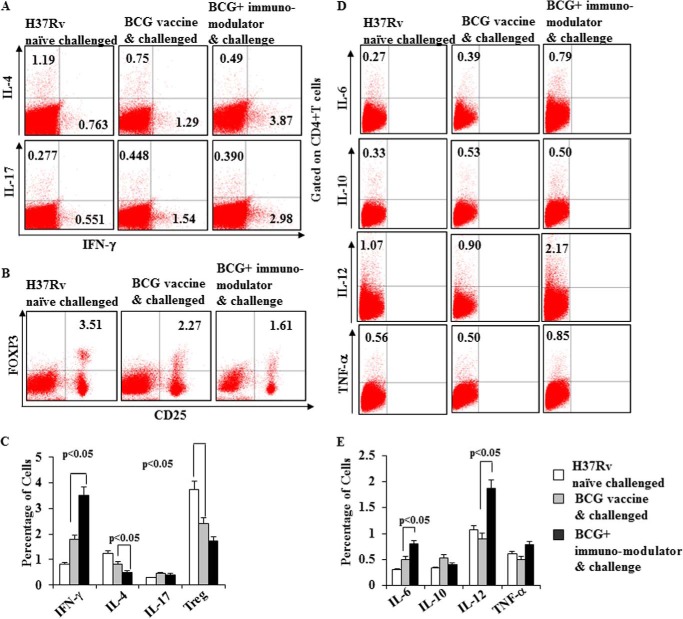
**BCG immunization plus immunomodulator treatment induces Th1 responses.** *A* and *B*, intracellular staining for IFN-γ, IL-4, IL-17, and FoxP3 (CD4^+^ CD25^+^) of CD4^+^ T lymphocytes isolated from the spleens of different groups (infected, BCG immunized, BCG immunized, and treated) of mice. *C* and *D*, intracellular cytokine staining for the production of IL-6, IL-10, IL-12, and TNF-α from different groups (infected, BCG-immunized, BCG-immunized, and treated) of mice. Results shown here are representative of three independent experiments.

To determine whether treatment with suplatast tosylate and D4476 affects immune responses in BCG immunized animals, we next quantified the frequencies of cytokine-producing T cells. Our results demonstrated that IFN-γ-producing CD4^+^ T cells were dramatically increased, whereas Th2 cytokine-producing cells were decreased in the animals treated with immunomodulators (Fig. 3, *A* and *C*, and supplemental Fig. S1). An opposite trend was observed in BCG-immunized animals in the absence of immunomodulators (Fig. 3, *A* and *C*, and supplemental Fig. S1). Interestingly, we were unable to detect changes in Th17 cytokine-producing cells in any experimental group (Fig. 3, *A* and *C*, and supplemental Fig. S1). Although Th17 responses promote vaccine efficacy (20, 21), such responses do not appear to be required for immune protection (22).

Treg cells play an important role in the pathogenesis of TB, and we, therefore, sought to determine the status of Treg cells in these animals. We observed an abrupt decrease of CD4^+^CD25^+^FoxP3^+^ T cells in BCG-vaccinated animals treated with immunomodulators (Fig. 3, *B* and *C*). We also determined other host-protective cytokines, including IL-6, IL-12, and TNF-α. Previously, IL-6 and TNF-α have been implicated in host resistance against *M. tuberculosis* (23). IL-12 is a Th1-inducing cytokine, and genetic deficiency in IL-12 or its signaling components causes profound susceptibility to *M. tuberculosis* infection. We found that IL-6-, IL-12-, and TNF-α-producing cells were significantly increased in BCG-immunized mice treated with immunomodulators (Fig. 3, *D* and *E*). We also examined IL-10 production but were unable to detect any differences (Fig. 3, *D* and *E*), which is in agreement with a previous report that IL-10^−/−^ animals are as susceptible as wild type littermates to *M. tuberculosis* infection (24).

##### Enhanced Central Memory T Cell Responses Induced by Immunomodulators Protect Animals from M. tuberculosis Infection

**FIGURE 4.**
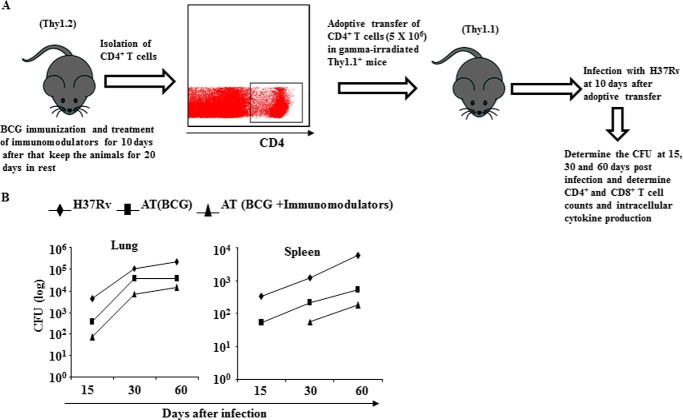
**T lymphocytes from mice immunized with BCG and treated with immunomodulators can adoptively transfer protective immunity against *M. tuberculosis* infection.** *A*, diagrammatic sketch of adaptive transfer of CD4^+^ T cells from Thy1.2^+^ donor mice to γ-irradiated Thy1.1^+^ mice. Vaccinated mice were used for CD4^+^ T cell isolation before adoptive transfer. *B*, after immunization Thy1.1^+^ mice were treated daily with D4476 (TGFβRI inhibitor) and/or suplatast tosylate (Th2 inhibitor) at 16 nmol/g of body weight for a total of 10 days and after a 20-day rest period T lymphocytes were isolated from lymph node and injected intraperitoneally (5 × 10^6^ cells) to γ-irradiated Thy1.2^+^ mice before aerosol challenge with a low dose of *M. tuberculosis* (H37Rv) (∼100 cfu). Bacterial burdens (cfu) were measured in lungs and spleens at 15, 30, and 60 days post infection. Data represent the mean ± S.D. values of four mice per group per time point, and the experiment was repeated twice. *AT*, adoptive transfer.

To provide further evidence for a critical role of Tcm generated by treatment with immunomodulators during BCG vaccination in resistance to *M. tuberculosis* infection, we immunized Thy1.2 congenic mice and treated these animals with suplatast tosylate and D4476, isolated CD4^+^ T cells, and adoptively transferred them into irradiated Thy1.1 congenic animals followed by infection with H37Rv (Fig. 4*A*). 15, 30, and 60 days after infection we measured the bacterial burden in the lungs and spleens of the mice. Interestingly, we found that recipient animals receiving cells from drug-treated mice displayed significantly reduced bacterial burden than recipients receiving cells from untreated mice (Fig. 4*B*). These findings suggested that Tcm cells generated by treatment with immunomodulators during BCG vaccination confer protective immunity against *M. tuberculosis* infection.

##### Immunomodulators Alter the Distribution of Cytokine-producing CD4^+^ T Cell Subsets in BCG-immunized Mice

**FIGURE 5.**
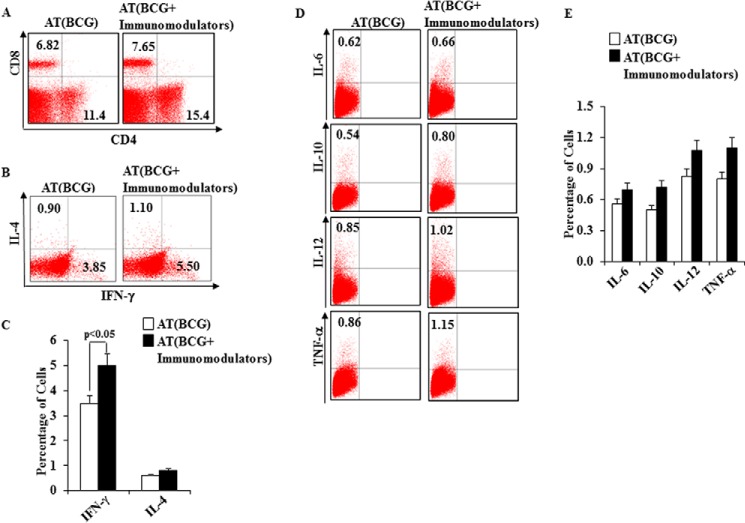
**Frequency of T cells and cytokine-producing cells in mice adoptively transferred with T cells from animals immunized with BCG and treated with immunomodulators.** *A*, profile of CD4^+^ and CD8^+^ T cell counts at 60 days post infection of mice that received T cells from BCG-immunized and immunomodulator-treated mice. *B* and *C*, intracellular staining for IFN-γ and IL-4 of CD4^+^ T lymphocytes isolated from the spleens of different groups of adoptively transferred mice. *D* and *E*, frequency of IL-6, IL-10, IL-12, and TNF-α intracellular cytokine-producing cells of different groups of adoptively transferred mice. Results shown here are representative of three independent experiments. *AT*, adoptive transfer.

It is well established that the proinflammatory cytokines IL-6 and TNF-α and Th1-associated cytokines play a central role in host protection against *M. tuberculosis* infection. Therefore, we measured these cytokines in animals that were adoptively transferred with memory T cells and protected from *M. tuberculosis* infection (Fig. 5*A* and supplemental Fig. 2). We observed that IFN-γ levels were much higher in the animals that received CD4^+^ T cells from drug-treated compared with untreated mice, whereas IL-4 levels were very similar (Fig. 5*B*). We also determined levels of the T cell-polarizing cytokines IL-6, IL-12, IL-10, and TNF-α. We found that IL-6, IL-12, and TNF-α levels were higher in the recipient animals that received cells from drug-treated mice (Fig. 5, *D* and *E*, and supplemental Fig. 1).

## DISCUSSION

Despite enormous efforts, BCG is currently the only available vaccine for prevention of TB. Thus far, none of the vaccine candidates tested in clinical trials have surpassed the efficacy of BCG (25). Currently, there are 11 vaccine candidates that are being tested in clinical trials, and 7 of these are subunit booster vaccines (26, 27). Because attenuated microorganisms, at least for bacterial diseases, have been highly successful as vaccines compared with subunit vaccines, recombinant BCG strains might represent promising vaccine candidates. However, none of the recombinant strains tested thus far have exceeded the vaccine efficacy of the parental BCG strain (28, 29). Nevertheless, two recombinant BCG vaccines are being tested in vaccine trials. Both of these recombinants, an Ag85-recombinant and a listeriolysin-recombinant, elicited superior vaccine efficacy than BCG in preclinical animal models (30). Ag85 is an antigenic protein from *M. tuberculosis* and may contribute to host-protective immune responses, and hence, a booster dose with this antigen may be helpful (31, 32). On the other hand, listeriolysin is a membrane-perforating protein responsible for cytosolic translocation of *Listeria* pathogens, which facilitates MHC class I-restricted antigen processing and induction of cytolytic CD8^+^ T cells (30). Thus, a BCG recombinant containing listeriolysin would be expected to facilitate escape of the organism (BCG) or leakage of its proteins into the cytosol and induce enhanced CD8^+^ T cell activation (33). We and other research groups have previously shown that *M. tuberculosis* itself can translocate from endosomal compartments to the cytosol (34, 35) and induce CD8^+^ T cell responses. Therefore, introduction of listeriolysin in BCG would induce immune responses that more closely mimic those induced by *M. tuberculosis*. Furthermore, listeriolysin may induce apoptosis in infected cells, which would facilitate cross-presentation of *M. tuberculosis* antigens on MHC class I to CD8^+^ T cells. Such cross-presentation may also induce Th1 and Th17 responses, which are known to contribute to host protection (15, 20). Although the role of CD8^+^ T cells in immune protection to human TB is still unclear, *in vitro* studies have suggested that they recognize heavily infected cells, which is consistent with a role of CD8^+^ T cells in immune surveillance against TB (36, 37). Targeting these cells may provide a means to prevent reactivation of latent infection (38).

Recently, we and other groups of investigators have shown that Th1 and to a lesser extent Th17 cells play important roles in host protection against TB (15, 20). Although BCG effectively induces Th1 responses, it has very limited efficacy against pulmonary TB. Thus, two possibilities might explain the limited vaccine efficacy of BCG. First, BCG may fail to induce an immune response that is critical for optimal host protection. Second, BCG may induce additional immune responses that hinder host-protective immune responses. Recently, we have shown that inhibition of Th2 and Treg cell differentiation by small molecules greatly enhances host-protective immunity (15). BCG similarly induces Th2 and Treg cell responses, and thus may hinder host-protective Th1 and/or Th17 cell responses. Our results reported here have demonstrated that simultaneous inhibition of Th2 cells and Tregs by small molecules potently enhances BCG-induced vaccine efficacy. Vaccine efficacy is governed by the capacity to induce Tcm cells. Indeed, our findings showed that simultaneous inhibition of Th2 and Tregs cells by small molecules enhances host-protective Tcm cell responses, which correlated with enhanced host protection. Furthermore, these observations suggest that Th2 cells and Tregs not only counterbalance host-protective immune responses that facilitate disease progression but also inhibit Tcm, resulting in reduced vaccine efficacy. Our earlier studies showed that simultaneous inhibition of Th2 cells and Tregs during *M. tuberculosis* infection promotes host-protective immunity, but this was not sufficient to completely eradicate the harbored *M. tuberculosis* organisms (15). Therefore, a recombinant BCG vaccine that can induce both Th1 and Th17 responses along with inhibition of Th2 and Treg responses should provide a more optimal vaccine strategy. This hypothesis will be explored in our future investigations.

Although BCG vaccination is ineffective against adult pulmonary TB, it is effective in protecting young children against meningeal and disseminated TB. As huge numbers of people have already received BCG, a strategy that selectively enhances Th1 and Th17 responses is highly desired. Our findings together with published studies suggest that BCG vaccine along with inhibition of Th2 and Treg cell differentiation may provide such a strategy.

## Supplementary Material

Supplemental Data

## Footnotes

3 The abbreviations used are:

TB: tuberculosis
BCG: Bacillus Calmette-Guerin
Tem: T cell effector memory
Tcm: central memory T
Th1: T helper 1
Treg: T regulatory
TGFβRI: TGFβ receptor inhibitor
D4476: 4-[4-(2,3-dihydro-1,4-benzodioxin-6-yl)-5-(2-pyridinyl)-1H-imiodazol-2-yl]benzamide.
