# Supplementary material for: Simultaneous Inhibition of T Helper 2 and T Regulatory Cell Differentiation by Small Molecules Enhances Bacillus Calmette-Guerin Vaccine Efficacy against Tuberculosis
Source: J Biol Chem. 2014 Oct 14;289(48):33404–11. doi: 10.1074/jbc.M114.600452 (PMC4246096; doi:10.1074/jbc.M114.600452)
Supplement: Supplemental Data [file supp_289_48_33404__index.html]

Simultaneous inhibition of T helper 2 and T regulatory cell differentiation by small molecules enhances Bacillus Calmette-Guerin vaccine efficacy against tuberculosis — Simultaneous Inhibition of T Helper 2 and T Regulatory Cell Differentiation by Small Molecules Enhances Bacillus Calmette-Guerin Vaccine Efficacy against Tuberculosis — Improving the Efficacy of BCG with Immunomodulators — Supplemental Data 

# Simultaneous Inhibition of T Helper 2 and T Regulatory Cell Differentiation by Small Molecules Enhances Bacillus Calmette-Guerin Vaccine Efficacy against Tuberculosis

## Supplemental Data

**Files in this Data Supplement:**

- Supplementary Figures.pdf (.pdf, 129 KB) - Supplementary Figures
