# Supplementary material for: Simultaneous Inhibition of T Helper 2 and T Regulatory Cell Differentiation by Small Molecules Enhances Bacillus Calmette-Guerin Vaccine Efficacy against Tuberculosis
Source: J Biol Chem. 2014 Oct 14;289(48):33404–11. doi: 10.1074/jbc.M114.600452 (PMC4246096; doi:10.1074/jbc.M114.600452)
Supplement: Supplemental Data [file supp_M114.600452_jbc.M114.600452-1.pdf]

Simultaneous inhibition of T helper 2 and T regulatory cell differentiation by small molecules enhances Bacillus Calmette-Guerin vaccine efficacy against tuberculosis

Debapriya Bhattacharya[1#], Ved Prakash Dwivedi[1#], Santosh Kumar[1], Madhava C Reddy[2], Luc Van Kaer[3], Prashini Moodley[1], Gobardhan Das[1, 4,*]

[1]Laboratory Medicine and Medical Sciences, College of Health Sciences, University of Kwazulu Natal, Durban, South Africa.

[2]Yogi Vemana University, Kadapa- 516003, India

[3]Department of Pathology, Microbiology and Immunology, Vanderbilt University School of Medicine, Nashville, TN, USA.

[4]Special Centre for Molecular Medicine, Jawaharlal Nehru University, New Delhi, India

[#]These authors contributed equally to this manuscript.

*Address for correspondence:

Prof. Gobardhan Das, Ph.D.
Special Centre for Molecular Medicine,
Jawaharlal Nehru University,
New Delhi, India
&
Laboratory Medicine and Medical Sciences
College of Health Sciences
UKZN
Durban, South Africa
E-mail: gobardhan.das07@gmail.com

Key Words: *Mycobacterium tuberculosis* (*M. tb*), T helper cell response, Immunomodulation, Vaccine.

Running title: Improving the efficacy of BCG with immunomodulators.

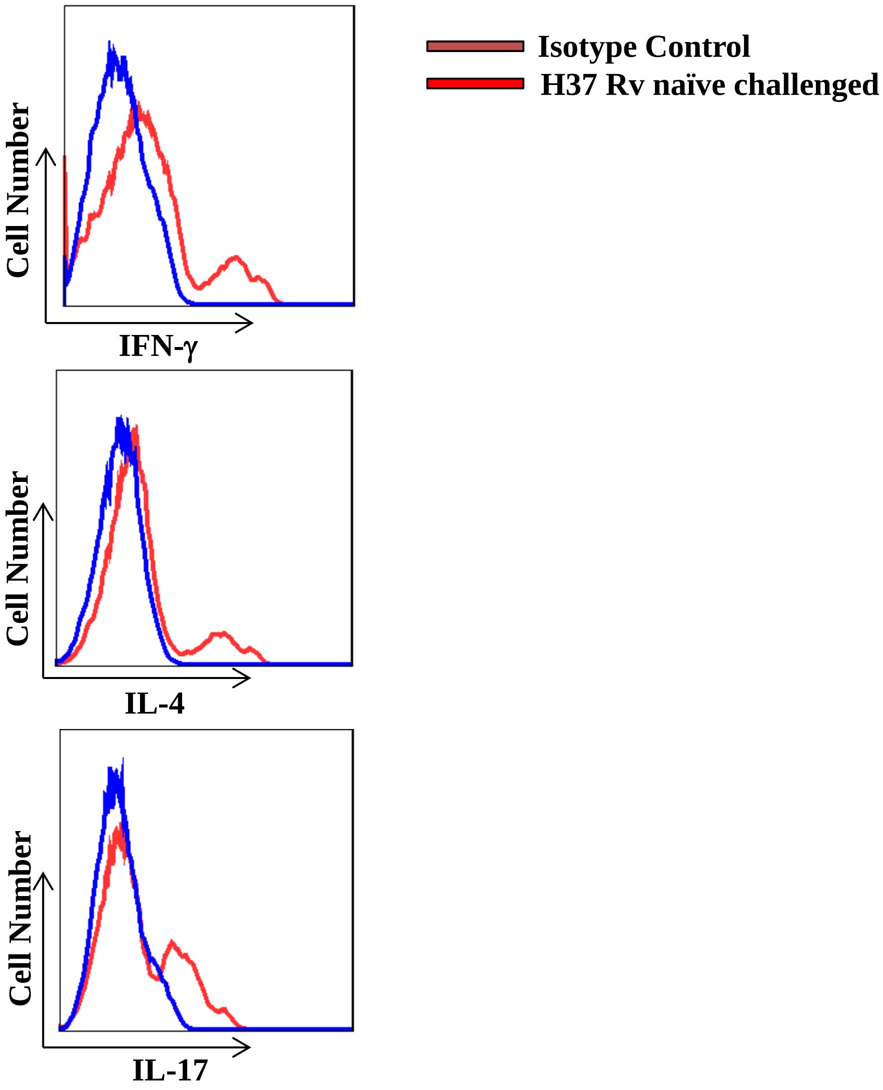

Supplementary Figure 1 (S1). Isotype control for intracellular cytokine staining (IFN- γ , IL-4, IL-17). For the Intracellular cytokine assay, we have used isotype control to compensate the background.

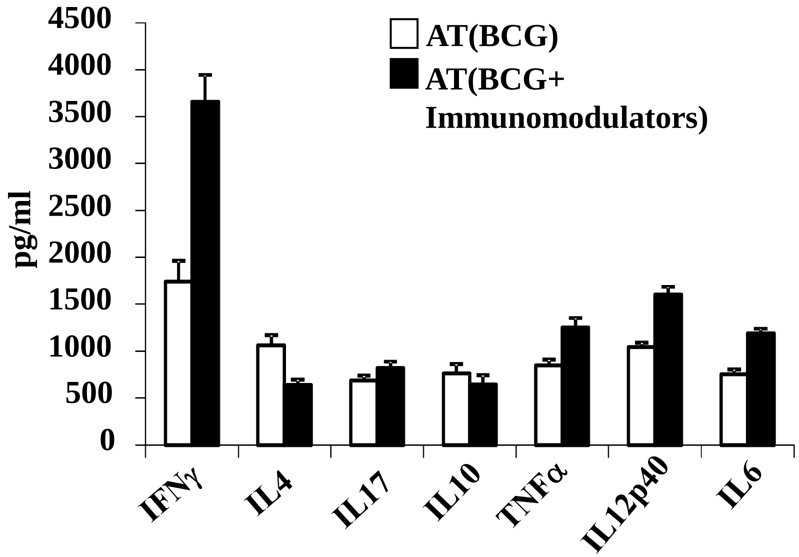

Supplementary Figure 2 (S2). Adoptively transferred mice that received CD4+ T cells from BCG-vaccinated and immunomodulator-treated mice show improved host-protective cytokine responses against M. tb challenge. For antigen-specific responses, single cell suspensions were obtained from spleen of the indicated mice at 60 days after H37Rv challenge. RBCs were lysed with Tris/NH4Cl, and the cells were washed extensively. Single cell suspensions (5 × 106 cells/well of 24-well plates) were stimulated with CSA of H37Rv (50 µg/ml) for 48 h and supernatants were collected for cytokine assay by Luminex microbead-based multiplexed assay. AT, adoptive transfer.

Fig S2. Bhattacharya, D. et al
